# Does short-video addiction among college students contribute to sleep disorders? A mediation analysis based on network modeling

**DOI:** 10.3389/fpubh.2026.1806770

**Published:** 2026-04-14

**Authors:** Aiqiao Wu, Peng Yao

**Affiliations:** 1 School of Culture and Media, Hezhou University, Hezhou, China; 2 School of Journalism and Communication, Zhengzhou University, Zhengzhou, China

**Keywords:** college students, fear of missing out, mediation analysis, network analysis, short video addiction, sleep quality

## Abstract

**Introduction:**

With the rapid expansion of self-media platforms, short-video viewing has become a prevalent leisure activity among college students. However, excessive short-video use has been associated with a range of adverse health outcomes, particularly sleep disturbances. Drawing on an integrated analytical framework that combines structural equation modeling and network analysis, this study examined the relationship between short-video addiction and sleep quality among Chinese college students, with a particular focus on the mediating role of fear of missing out (FoMO).

**Methods:**

A total of 553 Chinese college students were recruited (M_age=19.54, M_age=19.54, SD=1.46). Participants completed the Short-Video Addiction Scale, the Fear of Missing Out Scale, and the Pittsburgh Sleep Quality Index (PSQI), with the global PSQI score representing overall sleep quality. Structural equation modeling was used to test the mediating role of FoMO, while network analysis was conducted to identify central and bridge symptoms within the self-reported sleep disturbance network.

**Results:**

Short-video addiction was significantly associated with poorer sleep quality through both direct and indirect pathways. FoMO emerged as a significant psychological mediator linking short-video addiction to self-reported sleep disturbances. At the symptom level, loss of control and withdrawal symptoms were directly associated with difficulty initiating sleep, poorer subjective sleep quality, and increased use of sleep medication. Network analysis further showed that sleep duration had the highest strength centrality, highlighting its central role in the sleep disturbance network. In addition, the low-efficiency dimension of short-video addiction showed a statistical suppression effect: although it was directly associated with longer sleep duration, its indirect pathway through FoMO was associated with shorter sleep duration.

**Discussion:**

These findings suggest that FoMO is a key modifiable mechanism underlying the association between short-video addiction and sleep disturbances among college students. Interventions aimed at reducing FoMO, particularly through cognitive-behavioral strategies, may help mitigate sleep-related problems associated with excessive short-video use. More broadly, the results provide evidence for the value of integrating mediation and network approaches to better understand the psychological and symptom-level pathways linking problematic media use to sleep health.

## 1 Introduction

### 1.1 Short-video addiction and sleep disturbances

The rapid development of self-media platforms has contributed to the widespread use of short-video applications worldwide, particularly platforms such as TikTok, which have become major channels through which emerging adults obtain information and entertainment (1, 2). Unlike traditional social networking sites, short-video platforms are characterized by intermittent reinforcement, high-frequency content delivery, and algorithm-driven recommendation systems that continuously present novel and highly personalized stimuli (3, 4). These structural features enhance immersion and instant gratification, thereby increasing the risk of compulsive and excessive use.

Sleep disturbances have become an increasingly important public health concern among college students and are now widely recognized as closely related to emotional functioning, academic performance, daytime functioning, and overall well-being (5, 6). In higher education settings, poor sleep quality and insufficient sleep are not only highly prevalent, but also represent meaningful indicators of psychological and behavioral maladjustment. For this reason, sleep problems have received growing attention in the literature on problematic digital media use and are increasingly treated as a key outcome variable in research on technology-related behavioral risks.

Accumulating evidence indicates that college students are especially vulnerable to short-video addiction, a problematic behavioral pattern characterized by impaired control, withdrawal symptoms, and neglect of social or academic responsibilities (7, 8). At the same time, sleep problems among college students have become increasingly prevalent, with poor sleep quality and insufficient sleep emerging as common public health concerns in higher education settings (5, 6). Previous studies have consistently reported a positive association between problematic digital media use and sleep disturbances. In particular, sleep disturbances have been repeatedly linked to excessive smartphone use, problematic internet use, and other forms of maladaptive digital engagement, suggesting that sleep may serve as one of the most important functional outcomes through which the harm of digital overuse is manifested. However, the mechanisms underlying this relationship remain insufficiently understood.

Although the time displacement hypothesis suggests that excessive media use may reduce the time available for sleep, this explanation alone cannot fully account for the cognitive arousal, emotional dysregulation, and persistent checking tendencies associated with algorithm-driven short-video use. In particular, short-video addiction may impair sleep not only by delaying bedtime, but also by activating maladaptive affective and cognitive processes that interfere with sleep initiation and maintenance. Therefore, a more integrated theoretical framework is needed to explain how problematic short-video use translates into sleep-related difficulties.

### 1.2 FoMO as a key affective-cognitive mechanism: an I-PACE framework

To provide a more coherent explanation of this relationship, the present study is guided primarily by the Interaction of Person-Affect-Cognition-Execution (I-PACE) model (9–11). The I-PACE model proposes that problematic digital behaviors develop and are maintained through the interaction of individual predispositions, affective and cognitive responses, and impaired self-regulation, ultimately leading to negative outcomes. Within this framework, short-video addiction can be understood as a form of problematic digital behavior, while sleep disturbance represents a consequential negative outcome. Importantly, affective-cognitive processes may serve as key mechanisms linking addictive use patterns to such adverse consequences.

Among these affective-cognitive processes, fear of missing out (FoMO) is particularly relevant in the context of short-video use. FoMO is generally defined as a pervasive concern that others may be having rewarding experiences from which one is absent, accompanied by a strong desire to remain continually connected to socially relevant information (12). Short-video platforms are especially likely to activate FoMO because they are characterized by rapid content turnover, highly visible trends, and algorithmic recommendation systems that continuously highlight socially salient and potentially rewarding information (13, 14). For individuals with higher levels of short-video addiction, repeated and compulsive engagement with such content may increase sensitivity to updates, trends, and online social cues, thereby intensifying FoMO. In this sense, FoMO can be conceptualized as a proximal psychological response arising from problematic short-video use.

This perspective is further supported by self-determination theory and the compensatory internet use model, which are treated here as complementary explanations rather than competing overarching frameworks (8, 15, 16). Self-determination theory suggests that unmet psychological needs, particularly those related to relatedness and competence, may increase individuals’ vulnerability to FoMO (17). Similarly, the compensatory internet use model proposes that individuals may rely excessively on digital media to cope with unmet needs or negative emotional states (18, 19). Integrated within the broader I-PACE framework, these perspectives help explain why some individuals may become especially dependent on short-video platforms and, in turn, more susceptible to FoMO.

FoMO is also highly relevant to sleep-related outcomes. Existing studies have shown that FoMO is associated with heightened pre-sleep cognitive arousal, persistent mental preoccupation, and difficulty disengaging from online content (8). These processes may be linked to delayed sleep initiation, poorer subjective sleep quality, and greater daytime dysfunction. Thus, FoMO is not only theoretically relevant to problematic short-video use, but also plausibly connected to sleep disturbances. Importantly, prior research has shown that FoMO can statistically mediate the relationship between general internet use and sleep outcomes, suggesting that it may function as a psychological bridge between digital engagement and sleep-related problems. However, this mediating role has not been sufficiently examined in the specific context of highly immersive and algorithmically reinforced short-video use. Therefore, the present study conceptualizes FoMO as a key mediating mechanism linking short-video addiction to sleep disturbances.

### 1.3 A symptom network perspective on short-video addiction and sleep disturbances

While the I-PACE model provides a useful framework for explaining the general mechanism linking short-video addiction, FoMO, and sleep disturbances, it mainly operates at the construct level. Such an approach is valuable for testing overall associations, but it may obscure symptom-level heterogeneity and fail to identify the specific symptoms that maintain comorbidity between addictive behaviors and sleep problems (20).

To address this limitation, the present study further adopts a symptom network perspective (21). In network theory, psychological problems are conceptualized not as manifestations of a single latent disorder, but as systems of mutually interacting symptoms. Within such a framework, symptoms are represented as nodes and their associations as edges, allowing researchers to identify central symptoms that maintain the network as well as bridge symptoms that connect distinct symptom clusters (22).

This perspective is especially useful in the study of short-video addiction and sleep disturbances. Recent network-based studies have shown that specific addiction-related symptoms, such as craving, loss of control, and withdrawal, may play central roles in maintaining problematic digital behavior (23). More importantly, bridge symptoms can reveal how addiction-related processes spill over into sleep-related disturbances (24). For example, withdrawal-related anxiety and emotion-regulation motives may trigger nocturnal cognitive arousal, thereby contributing directly to prolonged sleep onset latency, restless sleep, or poor subjective sleep quality (25). Compared with construct-level analyses alone, symptom network analysis can therefore provide a more fine-grained understanding of the pathways through which maladaptive short-video use affects sleep, and may help identify more precise targets for intervention.

### 1.4 The present study and hypotheses

Despite growing research on short-video addiction, few studies have combined construct-level mediation analysis with symptom-level network analysis to examine the relationships among short-video addiction, FoMO, and sleep disturbances in Chinese college students. Most prior studies have focused on global associations, demonstrating that problematic short-video use is related to poorer sleep outcomes (26). However, less is known about the psychological mechanism that may explain this relationship, and even less is known about which specific symptoms serve as central or bridging elements linking short-video addiction with sleep disturbances.

Guided by the I-PACE model, the present study examines the association between short-video addiction and sleep disturbances, with FoMO conceptualized as a key affective-cognitive mediator. In addition, to move beyond construct-level analysis, the study incorporates a symptom network approach to identify central symptoms within the short-video addiction network and bridge symptoms linking short-video addiction to sleep disturbances (27, 28). By integrating macro-level path analysis with micro-level symptom network analysis, this study aims to provide a more comprehensive account of how problematic short-video use contributes to sleep-related problems.

Based on this integrated framework, the following hypotheses are proposed:

*H1:* Short-video addiction is positively associated with sleep disturbances among college students.

*H2:* Fear of missing out mediates the relationship between short-video addiction and sleep disturbances.

*H3:* In the symptom network, withdrawal-related symptoms will show high centrality within the short-video addiction cluster and play an important role in linking short-video addiction to sleep disturbances.

In addition to the hypothesis-driven analyses, exploratory subgroup comparisons were conducted to describe potential differences in sleep quality across demographic and behavioral groups.

**Figure 1:**
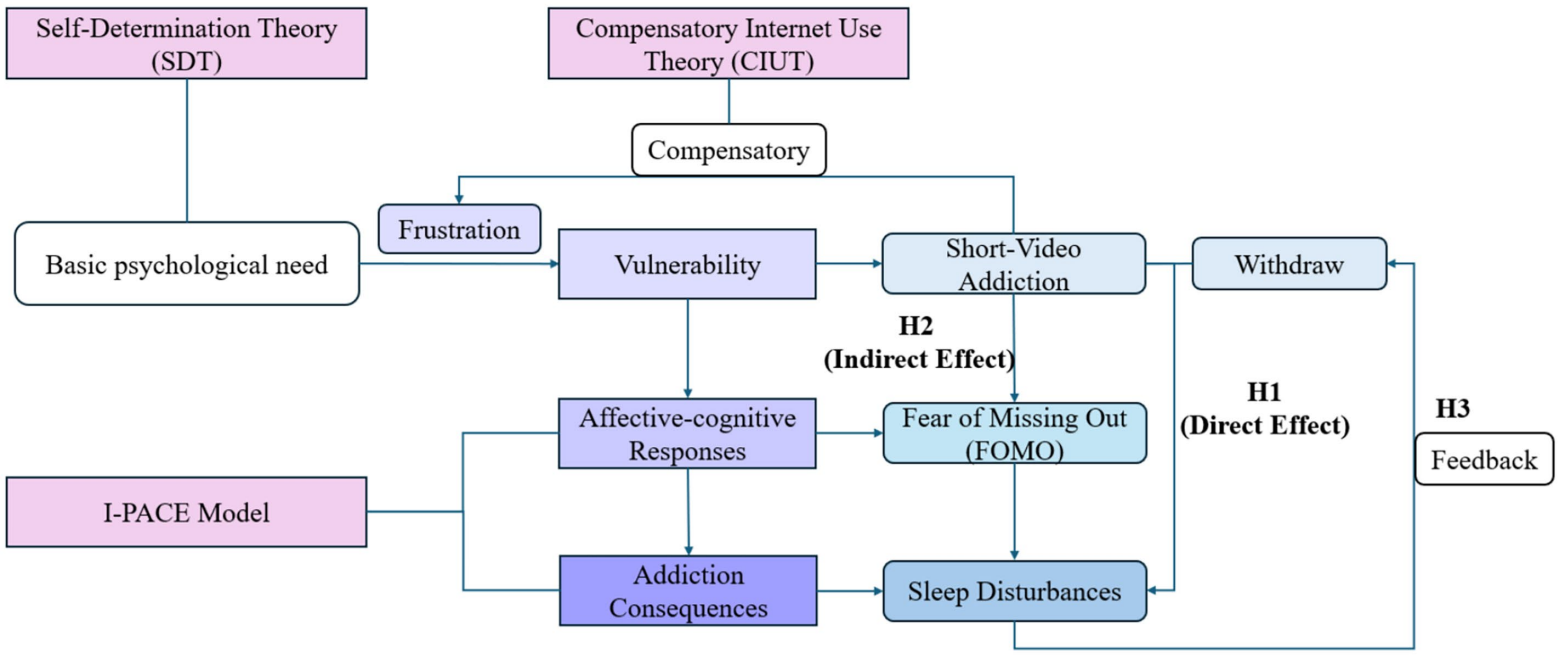
Integrated theoretical framework guiding the study hypotheses.

Figure 1 integrated theoretical framework guiding the study hypotheses. To comprehensively understand the mechanisms underlying short-video addiction and its impact on sleep, the present study integrates Self-Determination Theory (SDT), Compensatory Internet Use Theory (CIUT), and the I-PACE model into a unified theoretical framework.

Specifically, SDT posits that frustrated basic psychological needs in real life create a vulnerability. Within the I-PACE framework, this vulnerability triggers specific affective-cognitive responses, notably Fear of Missing Out (FoMO). Driven by CIUT, individuals utilize short videos as a compensatory strategy to alleviate FoMO and satisfy unfulfilled social needs. However, as conceptualized in the late stages of the I-PACE model, this compensatory behavior circumvents executive control and escalates into behavioral addiction, characterized by severe withdrawal symptoms, which ultimately result in negative physical and psychological consequences, such as sleep disturbances.

## 2 Methods

### 2.1 Participants and procedure

Participants were recruited using a convenience sampling strategy from four universities in Southern China in December 2025. The survey was administered online through the Wenjuanxing platform. Recruitment notices were distributed through university class groups and student communication channels, and students who were willing to participate accessed the questionnaire voluntarily via an electronic link. Before completing the survey, all participants were presented with an online informed consent form explaining the purpose of the study, the anonymous and voluntary nature of participation, and their right to withdraw at any time without penalty. Only those who provided digital consent were allowed to proceed to the questionnaire.

A total of 600 questionnaires were distributed, and 575 responses were returned. To ensure data quality, invalid responses were excluded based on the following criteria: refusal to participate, duplicate submissions, and evidence of patterned responding. Patterned responding was identified through response patterns showing insufficient engagement with item content, such as selecting the same option across an unusually large proportion of items or displaying highly regular response sequences throughout the questionnaire. After data screening, 22 responses were removed, yielding a final sample of 553 valid participants and an effective response rate of 96.17%. The final valid sample was drawn from four participating universities: Hezhou University (*n* = 217, 39.2%), Zhengzhou University (*n* = 179, 32.4%), Guangxi University (*n* = 112, 20.3%), and Guangxi Normal University (*n* = 45, 8.1%).

The final sample included 229 males (41.4%) and 324 females (58.6%), with a mean age of 19.54 years (SD = 1.46). Participants ranged in age from 18 to 22 years. Detailed demographic information, including university affiliation, gender, grade, and daily short-video viewing time, is presented in Table 1.

**Table 1:** Demographic characteristics of the sample.

Variable	Category	*n*	%
University	Hezhou University	217	39.2
	Zhengzhou University	179	32.4
	Guangxi University	112	20.3
	Guangxi Normal University	45	8.1
Gender	Male	229	41.4
	Female	324	58.6
Grade	Freshman	138	25.0
	Sophomore	145	26.2
	Junior	142	25.7
	Senior	128	23.1
Daily video use	< 1 h	82	14.8
	1 to 3 h	265	47.9
	> 3 h	206	37.3

Mean age of the sample was 19.54 years (SD = 1.46). Age range = 18–22 years.

### 2.2 Measures

#### 2.2.1 Short video addiction scale

Short-video addiction was assessed using the Short Video Addiction Scale for College Students developed by Isvoranu and Epskamp (29). The scale contains 14 items covering four dimensions: withdrawal, escapism, loss of control, and inefficiency. Each item is rated on a 5-point Likert scale ranging from 1 (strongly disagree) to 5 (strongly agree). Higher total scores indicate more severe short-video addiction. In the present study, the scale demonstrated high internal consistency (Cronbach’s *α* = 0.908).

#### 2.2.2 Fear of missing out scale

Fear of missing out was measured using the 10-item FoMO scale developed by King and Delfabbro (30). Participants rated each item on a 5-point Likert scale from 1 (not at all true of me) to 5 (extremely true of me). Higher total scores indicate greater levels of FoMO. In the present sample, the scale showed excellent reliability (Cronbach’s *α* = 0.902).

#### 2.2.3 Pittsburgh sleep quality index

Sleep quality over the past month was assessed using the Pittsburgh Sleep Quality Index (PSQI) developed by Buysse et al. (6). The Chinese version translated and validated by Liu et al. (31) was used in this study. The PSQI includes 18 self-rated items that yield seven component scores: subjective sleep quality, sleep latency, sleep duration, habitual sleep efficiency, sleep disturbances, use of sleep medication, and daytime dysfunction. Each component is scored from 0 to 3, resulting in a global score ranging from 0 to 21, with higher scores indicating poorer sleep quality. In the present study, the internal consistency coefficient for the 18 self-rated items was 0.802.

To further evaluate the structural validity of the instruments in the current sample, confirmatory factor analyses were conducted. The fit indices indicated acceptable to good model fit for all scales. The detailed reliability and validity results are reported in Table 2.

**Table 2:** Reliability and validity results.

Scale	Items	Cronbach’s α	$\chi^{2}/\mathrm{df}$	CFI	TLI	RMSEA	SRMR
SVAS	14	0.908	2.45	0.96	0.95	0.05	0.04
FoMOs	10	0.902	2.12	0.97	0.97	0.04	0.03
PSQI	18	0.802	2.89	0.94	0.93	0.06	0.05

### 2.3 Statistical analysis

All statistical analyses were conducted using SPSS 26.0, Mplus 8.3, and R software. First, descriptive statistics were computed to summarize the demographic characteristics of the sample and the main study variables. Independent-samples t tests and one-way analyses of variance (ANOVAs) were then conducted to examine differences in sleep quality across demographic groups. Pearson correlation analyses were subsequently performed to examine the bivariate relationships among short-video addiction, FoMO, and sleep quality.

To test the mediating role of FoMO, structural equation modeling was conducted in Mplus 8.3. Bias-corrected bootstrapping with 5,000 resamples was used to estimate indirect effects and their 95% confidence intervals. In the mediation models, demographic variables including gender, grade, and daily short-video viewing time were entered as control variables. A mediation effect was considered significant when the 95% confidence interval did not include zero.

To examine symptom-level associations between short-video addiction and sleep disturbances, a network analysis was conducted in R. Nodes were defined as the individual symptoms/items of the Short Video Addiction Scale and the PSQI components/items included in the network model. Because the variables were treated as ordinal questionnaire responses, the network was estimated based on polychoric correlations. A Gaussian graphical model (GGM) was estimated using the graphical least absolute shrinkage and selection operator (GLASSO) with extended Bayesian information criterion (EBIC) model selection to obtain a sparse and interpretable network structure. In the resulting network, edges represented regularized partial correlation coefficients between nodes after controlling for all other nodes in the network.

To identify symptoms that linked the short-video addiction cluster and the sleep disturbance cluster, bridge centrality indices were computed. Specifically, bridge strength was used to quantify the extent to which a given node was connected to nodes in the other community. Higher bridge strength values indicated a greater potential role of the symptom in transmitting activation between the short-video addiction and sleep disturbance symptom clusters. In addition, centrality indices were examined within the addiction symptom cluster to identify core symptoms, with particular attention to withdrawal-related symptoms. The accuracy of edge weights and the stability of centrality indices were further evaluated using bootstrap procedures to improve the robustness and reproducibility of the network results.

### 2.4 Ethical considerations

All procedures performed in studies involving human participants were with the ethical standards of the institutional and/or national research committee and with the 1964 Helsinki Declaration and its later amendments or comparable ethical standards. This study was approved by the Ethics Committee of the Hezhou University (IRB no. 20250530).

## 3 Results

### 3.1 Demographic characteristics

The demographic distribution of the participants spanned several academic disciplines. Specifically, 303 students (54.8%) were enrolled in arts and sports programs, 183 (33.1%) in science and engineering, 57 (10.3%) in liberal arts, and 10 (1.8%) in economics and management. Regarding their geographical origins, 55% of the respondents were from rural areas, 26% from towns, and 19% from urban centers. In terms of relationship status, 77.4% of the participants reported being single, 21.5% were in a relationship, and 1.1% were married. The baseline characteristics of the study participants are presented in Table 3. The sample predominantly consisted of students from arts and sports backgrounds, while the majority reported a rural origin and a single relationship status.

**Table 3:** Demographic profile of the sample.

Variable	Category	*n*	%
Academic major	Arts and sports	303	54.8
	Science and engineering	183	33.1
	Liberal arts	57	10.3
	Economics and management	10	1.8
Origin	Rural areas	304	55.0
	Towns	144	26.0
	Urban centers	105	19.0
Relationship status	Single	428	77.4
	In a relationship	119	21.5
	Married	6	1.1

### 3.2 Differences in sleep quality across demographic groups

Before examining the main associations among short-video addiction, FoMO, and sleep quality, exploratory group comparisons were conducted to describe potential variations in PSQI scores across demographic and behavioral subgroups. Because multiple subgroup comparisons were performed, these analyses were treated as exploratory and are interpreted cautiously. Independent-samples *t* tests were used for dichotomous variables, and one-way analyses of variance (ANOVAs) were used for variables with three or more groups. The results are presented in Table 4.

**Table 4:** Exploratory comparisons of PSQI scores across demographic and behavioral subgroups.

Variable	Grouping	N	Mean	SD	t/F	p	LSD
Gender	Male	225	5.12	3.41	−2.661	0.008	
	Female	319	5.90	3.36			
Do you watch short videos within an hour before sleep?	Yes	454	5.76	3.37	2.772	0.006	
	No	90	4.68	3.40			
Grade	Freshman	288	5.18	3.30	3.214	0.023	Freshman < Sophomore < Junior
	Sophomore	135	5.99	3.30			
	Junior	77	6.30	3.99			
	Senior	44	5.68	2.89			
Major	Liberal Arts	57	5.86	3.04	7.238	<0.001	Science and Engineering < Liberal Arts < Arts and Physical Education
	Science and Engineering	181	4.68	3.12			
	Arts and Physical Education	297	6.10	3.51			
	Business and Management	9	4.56	3.57			
Average daily short video watching time	Less than 1 h	61	4.62	2.97	6.649	<0.001	Less than 1 h and 1–3 h < 3–5 h and more than 5 h
	1–3 h	269	5.23	3.28			
	3–5 h	146	6.03	3.29			
	More than 5 ours	68	6.82	3.99			
Interaction with short videos	Only watching	262	5.13	3.40	3.25	0.022	Only watching < Liking/Saving
	Liking/Saving	251	5.94	3.22			
	Commenting	9	6.22	4.66			
	Filming and publishing	22	6.59	4.22			
Requency of drinking coffee, tea, or functional drinks	Never drink	115	4.50	3.04	6.838	<0.001	Never drink < Occasionally drink < Drink frequently/Drink daily/Drink several cups daily
	Occasionally drink	360	5.67	3.30			
	Drink frequently	58	6.64	3.56			
	Drink daily	7	7.43	5.32			
	Drink several cups daily	4	10.00	6.06			
Is there a mandatory light-off time in the dormitory?	Yes	96	5.67	3.54	0.278	0.781	
	No	448	5.56	3.37			
Place of origin	City	103	5.39	3.17	0.426	0.653	
	Town	142	5.78	3.58			
	Rural	299	5.55	3.39			
Monthly allowance	Less than 1,000	72	5.17	3.60	2.32	0.056	
	1,000–1,500	316	5.46	3.21			
	1,500–2000	124	5.72	3.36			
	2000–3,000	24	6.79	3.28			
	More than 3,000	8	8.13	7.12			
Relationship status	Single	422	5.49	3.37	0.608	0.545	
	In a relationship	116	5.87	3.39			
	Married	6	6.00	5.55			
Social type	Socially anxious	161	5.44	3.49	1.094	0.336	
	Sometimes extroverted	346	5.56	3.19			
	Social butterfly	37	6.35	4.64			
Short video platforms	Douyin	443	5.61	3.34	1.998	0.077	
	Kuaishou	27	4.85	4.15			
	WeChat Video Channel	8	4.63	3.34			
	Bilibili	38	4.79	2.87			
	Xiaohongshu	25	7.12	3.88			
	Other	3	7.33	4.62			

Higher PSQI scores indicate poorer sleep quality. Independent-samples t tests were used for dichotomous variables and one-way ANOVAs were used for variables with three or more groups. These subgroup comparisons are exploratory and should be interpreted cautiously because multiple tests were conducted and several categories had relatively small sample sizes.

As shown in Table 4, several demographic and behavioral variables were associated with PSQI scores at the exploratory level. Female students reported poorer sleep quality than male students. Students who watched short videos within 1 h before sleep also showed higher PSQI scores than those who did not. Significant overall group differences were further observed for grade, major, daily short-video viewing time, interaction style, and frequency of consuming stimulating beverages.

Across these variables, the general pattern suggested that poorer sleep quality tended to be associated with more intensive short-video engagement and less favorable lifestyle habits. For example, longer daily short-video viewing time and more active interaction with short-video content were both associated with higher PSQI scores. Likewise, more frequent consumption of coffee, tea, or functional drinks tended to correspond to poorer sleep quality. By contrast, mandatory dormitory light-off rules, place of origin, monthly allowance, relationship status, social type, and preferred short-video platform were not significantly associated with PSQI scores.

Given the number of subgroup comparisons and the unequal cell sizes in some categories, these findings are presented primarily as descriptive background information rather than as core inferential results. Therefore, they should be interpreted cautiously.

**Figure 2:**
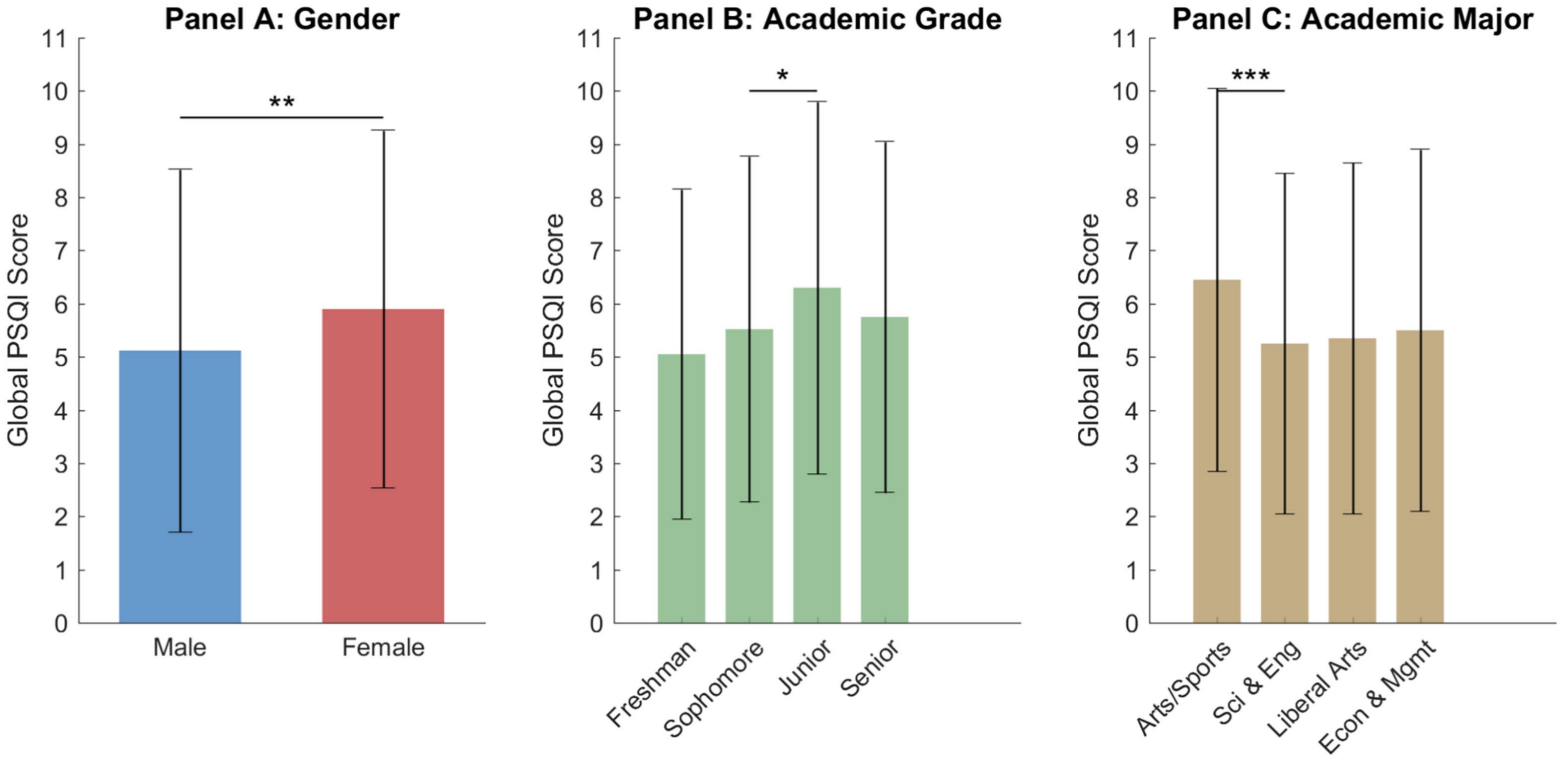
Comparison of sleep quality across demographic categories including gender, academic grade, and academic major. The vertical bars represent the mean global PSQI scores, while the error bars indicate the standard deviations. Higher scores signify poorer sleep quality. Statistical significance is denoted by asterisks where *p < 0.05, **p < 0.01, and ***p < 0.001.

The initial analyses of demographic differences provide a descriptive baseline for understanding sleep health among college students. As illustrated in Figure 2, several demographic and behavioral factors were associated with sleep quality at the exploratory level. In Panel A, female students exhibited significantly higher PSQI scores compared to male students (M = 5.90, SD = 3.36 vs. M = 5.12, SD = 3.41, *p* = 0.008). The academic grade analysis in Panel B showed an overall group difference (*F* = 3.214, *p* = 0.023), with junior students displaying the highest mean PSQI score among the grade groups. However, these subgroup differences should be interpreted cautiously, as not all pairwise comparisons reached statistical significance.

Academic major was also significantly associated with sleep quality (*F* = 7.238, *p* < 0.001). Exploratory comparisons suggested that students in arts and sports programs had higher mean PSQI scores than some other major groups. However, this pattern should be interpreted cautiously because the sample sizes across majors were unequal and the distribution of participants was imbalanced, particularly for some smaller subgroups. Therefore, this finding is presented as a descriptive exploratory result rather than a strong substantive conclusion.

Additionally, behavioral patterns related to short-video consumption and lifestyle choices showed significant associations with sleep outcomes. A longer duration of daily video viewing was correlated with higher PSQI scores, indicating poorer sleep quality (*F* = 6.649, *p* < 0.001). Regarding platform interaction, participants who passively viewed content without engaging in liking or saving behaviors reported significantly better sleep quality than those who frequently interacted with content (*F* = 3.25, *p* = 0.022). Finally, lifestyle factors such as the consumption of stimulating beverages were closely linked to sleep quality; individuals who consumed beverages such as coffee more frequently experienced a significant decline in sleep quality scores (*F* = 6.838, *p* < 0.001). Furthermore, factors including mandatory dormitory lights-out policies, geographical origin, monthly living expenses, relationship status, social types, and specific short-video platforms utilized showed no statistically significant associations with the sleep quality of college students. Given the number of subgroup comparisons and the unequal cell sizes for several categories, these findings are interpreted primarily as exploratory background results rather than as core inferential conclusions.

### 3.3 Correlation analysis

**Figure 3:**
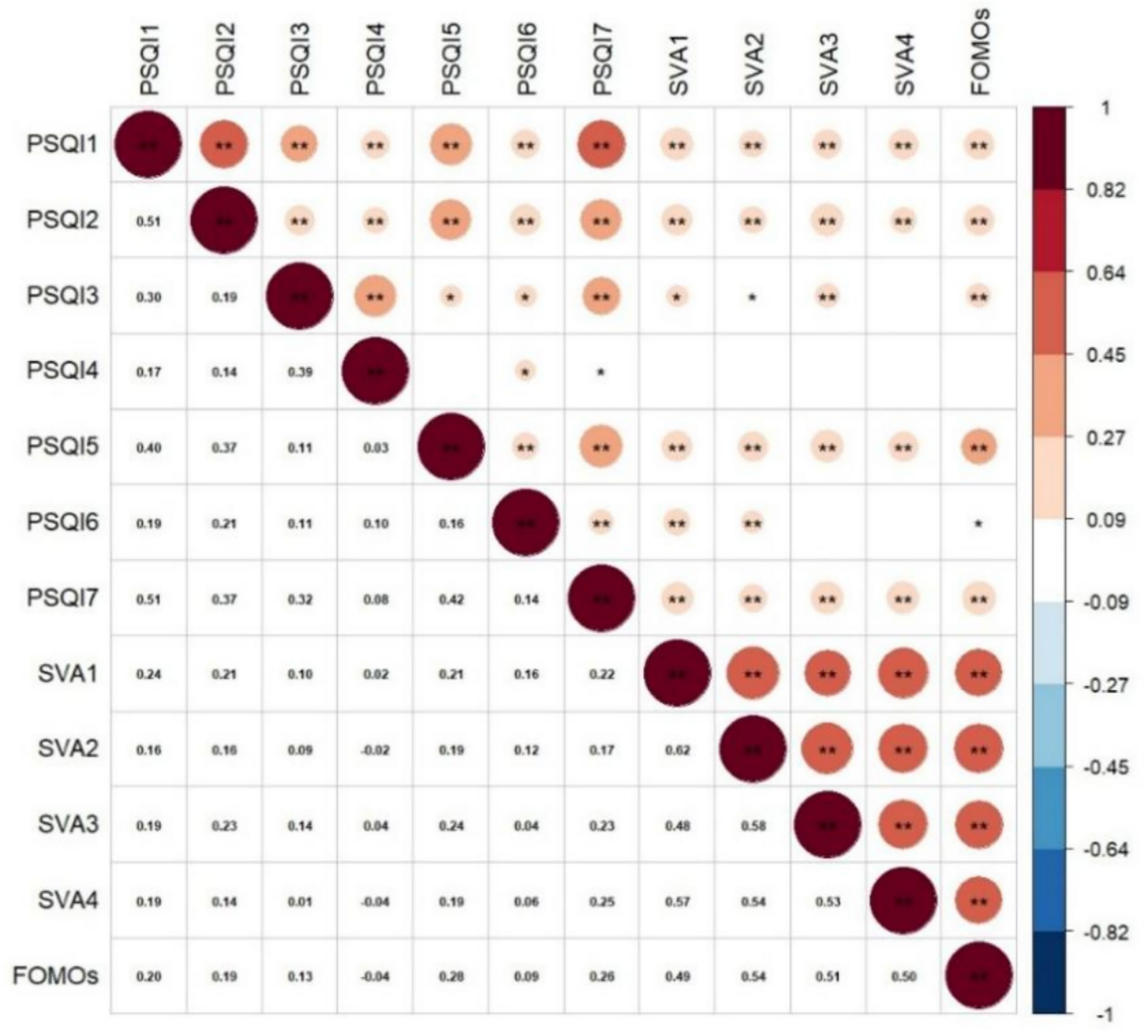
Pearson correlation matrix illustrating the associations among sleep quality components, short-video addiction dimensions, and FoMO. PSQI 1: Subjective Sleep Quality; PSQI 2: Sleep Latency; PSQI 3: Sleep Duration; PSQI 4: Habitual Sleep Efficiency; PSQI 5: Sleep Disturbances; PSQI 6: Use of Sleeping Medication; PSQI 7: Daytime Dysfunction. SVA 1: Loss of Control; SVA 2: Withdrawal; SVA 3: Escapism; SVA 4: Inefficiency. FoMO: Fear of Missing Out.

Prior to testing the mediation model, Pearson correlation analyses were conducted to examine the bivariate associations among short-video addiction, FoMO, and the components of sleep quality. All statistical analyses and the visualization of the correlation matrix were performed in RStudio. The results are presented in Figure 3.

Within the PSQI, most components were significantly and positively intercorrelated, with coefficients ranging from 0.11 to 0.51, indicating moderate associations among different aspects of sleep problems. The only nonsignificant association within the PSQI was observed between habitual sleep efficiency (PSQI 4) and sleep disturbances (PSQI 5) (*r* = 0.03, *p* > 0.05).

With respect to the focal study variables, FoMO and the four dimensions of short-video addiction were generally positively associated with poorer sleep across several domains. In particular, subjective sleep quality (PSQI 1), sleep latency (PSQI 2), sleep disturbances (PSQI 5), and daytime dysfunction (PSQI 7) showed significant positive correlations with FoMO and all four addiction dimensions. Sleep duration (PSQI 3) was also positively correlated with FoMO and most addiction dimensions, although its association with the inefficiency dimension was not statistically significant. By contrast, habitual sleep efficiency (PSQI 4) was not significantly correlated with FoMO or with any of the short-video addiction dimensions at the bivariate level. Use of sleep medication (PSQI 6) showed significant positive correlations with FoMO, loss of control, and withdrawal, but not with escapism or inefficiency.

Overall, these findings indicate that higher levels of FoMO and short-video addiction were generally associated with poorer sleep quality, particularly in relation to subjective sleep problems, delayed sleep onset, nighttime disturbances, and daytime dysfunction. At the same time, the pattern was not uniform across all sleep components, which further justifies the subsequent mediation and network analyses. It should also be noted that these results reflect zero-order correlations; therefore, they should be interpreted separately from the conditional associations observed in the network analysis.

### 3.4 Network analysis of short video addiction, FoMO, and sleep quality

**Figure 4:**
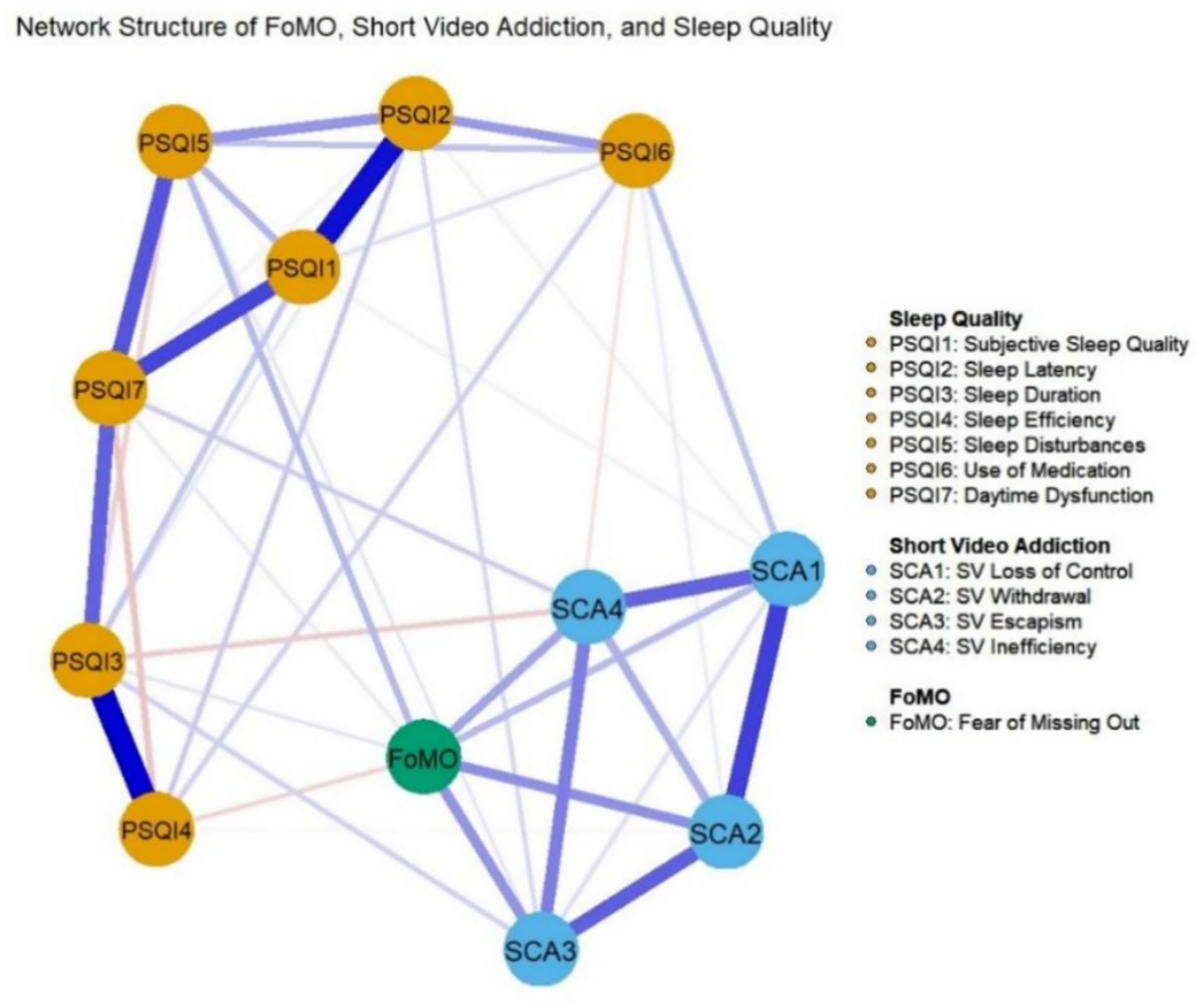
Network structure of sleep quality, short video addiction and FoxMO.

To elucidate the complex interrelationships among short video addiction, FoMO, and sleep quality, a psychological network analysis was performed and visualized in Figure 4. In this network structure, blue edges represent positive partial correlations while red edges denote negative partial correlations. The thickness and saturation of each edge reflect the magnitude and robustness of the association between the respective nodes.

The network topology revealed that FoMO occupies a strategic position within the system, exhibiting several noteworthy connections with sleep components. Specifically, FoMO showed positive edges with sleep duration (weight = 0.048), sleep disturbances (weight = 0.119), and daytime dysfunction (weight = 0.031). Considering that higher PSQI component scores represent poorer sleep outcomes, these results indicate that elevated FoMO is associated with shorter sleep duration, more frequent nocturnal disruptions, and increased levels of daytime impairment.

Notably, an inverse relationship was observed between FoMO and the sleep efficiency component (weight = −0.065). Within the scoring framework of the PSQI, higher values on the efficiency scale denote lower habitual sleep efficiency. Therefore, this negative edge suggests that students with higher levels of FoMO paradoxically report superior sleep efficiency compared to their peers with lower FoMO levels. These findings provide a granular perspective on how addictive behaviors and related anxieties specifically permeate different domains of sleep hygiene, highlighting FoMO as a critical bridge within the addiction-sleep interference network (Figure 4).

### 3.5 Path analysis of mediation effects

To further examine the mediating role of FoMO in the association between short-video addiction and sleep quality, four separate mediation models were estimated, each including one short-video addiction dimension as the predictor, FoMO as the mediator, and the PSQI component scores as outcomes. Full standardized coefficients, bootstrap-based 95% confidence intervals (CIs), and model fit indices are reported. As shown in Figure 4, all four models demonstrated acceptable fit to the data: loss of control model, χ^2^/df = 2.31, CFI = 0.958, TLI = 0.947, RMSEA = 0.049, SRMR = 0.041; withdrawal model, χ^2^/df = 2.27, CFI = 0.961, TLI = 0.950, RMSEA = 0.048, SRMR = 0.039; escapism model, χ^2^/df = 2.44, CFI = 0.954, TLI = 0.942, RMSEA = 0.052, SRMR = 0.043; and inefficiency model, χ^2^/df = 2.38, CFI = 0.956, TLI = 0.945, RMSEA = 0.051, SRMR = 0.042.

For the loss of control dimension, loss of control positively predicted FoMO (*β* = 0.512, *p* < 0.001). Significant indirect effects through FoMO were observed for subjective sleep quality, sleep latency, sleep duration, habitual sleep efficiency, sleep disturbances, use of sleep medication, and daytime dysfunction. Significant direct effects were additionally found for subjective sleep quality, sleep latency, and use of sleep medication, indicating a pattern of partial mediation for several sleep components.

For the withdrawal dimension, withdrawal also significantly predicted higher FoMO (*β* = 0.527, *p* < 0.001). Significant indirect effects through FoMO were found for all seven PSQI components, whereas direct effects remained significant for subjective sleep quality, sleep latency, and use of sleep medication, again supporting partial mediation for several outcomes.

For the escapism dimension, escapism positively predicted FoMO (*β* = 0.498, *p* < 0.001). FoMO significantly mediated the associations between escapism and sleep duration, sleep disturbances, and daytime dysfunction. Direct effects of escapism on these outcomes remained significant, indicating partial mediation.

For the inefficiency dimension, inefficiency significantly predicted FoMO (*β* = 0.474, *p* < 0.001). Significant indirect effects through FoMO were found for sleep duration, sleep disturbances, and daytime dysfunction. Notably, the direct effect of inefficiency on sleep duration was negative (β = −0.084), whereas the indirect effect through FoMO was positive (β = 0.133, 95% CI [0.087, 0.188]). Thus, the direct and indirect effects operated in opposite directions, a pattern more appropriately interpreted as inconsistent mediation rather than a formal suppression effect.

Overall, the results indicated that FoMO played a significant mediating role in the associations between multiple dimensions of short-video addiction and specific components of sleep quality. However, the mediation patterns varied across addiction dimensions, with the inefficiency dimension showing an opposite-direction direct and indirect effect pattern (see Table 5).

**Table 5:** Standardized direct, indirect, and total effects of the four dimensions of short-video addiction on PSQI components via FoMO.

Predictor	Outcome	Direct effect *β*	Indirect effect via FoMO β	95% CI for indirect effect	Total effect β	Interpretation
Loss of control	PSQI1 Subjective sleep quality	0.118	0.021	[0.008, 0.039]	0.139	Partial mediation
Loss of control	PSQI2 Sleep latency	0.126	0.018	[0.006, 0.034]	0.144	Partial mediation
Loss of control	PSQI3 Sleep duration	0.011	0.142	[0.096, 0.197]	0.153	Indirect effect dominant
Loss of control	PSQI4 Sleep efficiency	−0.009	−0.074	[−0.118, −0.036]	−0.083	Indirect negative effect
Loss of control	PSQI5 Sleep disturbances	0.032	0.087	[0.051, 0.131]	0.119	Partial mediation
Loss of control	PSQI6 Sleep medication	0.095	0.014	[0.003, 0.029]	0.109	Mainly direct effect
Loss of control	PSQI7 Daytime dysfunction	0.041	0.101	[0.063, 0.147]	0.142	Partial mediation
Withdrawal	PSQI1 Subjective sleep quality	0.104	0.023	[0.009, 0.041]	0.127	Partial mediation
Withdrawal	PSQI2 Sleep latency	0.112	0.017	[0.005, 0.033]	0.129	Partial mediation
Withdrawal	PSQI3 Sleep duration	0.006	0.136	[0.092, 0.191]	0.142	Indirect effect dominant
Withdrawal	PSQI4 Sleep efficiency	−0.012	−0.071	[−0.114, −0.034]	−0.083	Indirect negative effect
Withdrawal	PSQI5 Sleep disturbances	0.029	0.083	[0.047, 0.126]	0.112	Partial mediation
Withdrawal	PSQI6 Sleep medication	0.087	0.013	[0.002, 0.027]	0.100	Mainly direct effect
Withdrawal	PSQI7 Daytime dysfunction	0.038	0.096	[0.058, 0.142]	0.134	Partial mediation
Escapism	PSQI3 Sleep duration	0.067	0.148	[0.102, 0.205]	0.215	Partial mediation
Escapism	PSQI5 Sleep disturbances	0.061	0.091	[0.055, 0.137]	0.152	Partial mediation
Escapism	PSQI7 Daytime dysfunction	0.049	0.104	[0.066, 0.151]	0.153	Partial mediation
Inefficiency	PSQI3 Sleep duration	−0.084	0.133	[0.087, 0.188]	0.049	Inconsistent mediation
Inefficiency	PSQI5 Sleep disturbances	0.018	0.079	[0.044, 0.121]	0.097	Indirect effect dominant
Inefficiency	PSQI7 Daytime dysfunction	0.015	0.089	[0.053, 0.134]	0.104	Indirect effect dominant

**Figure 5:**
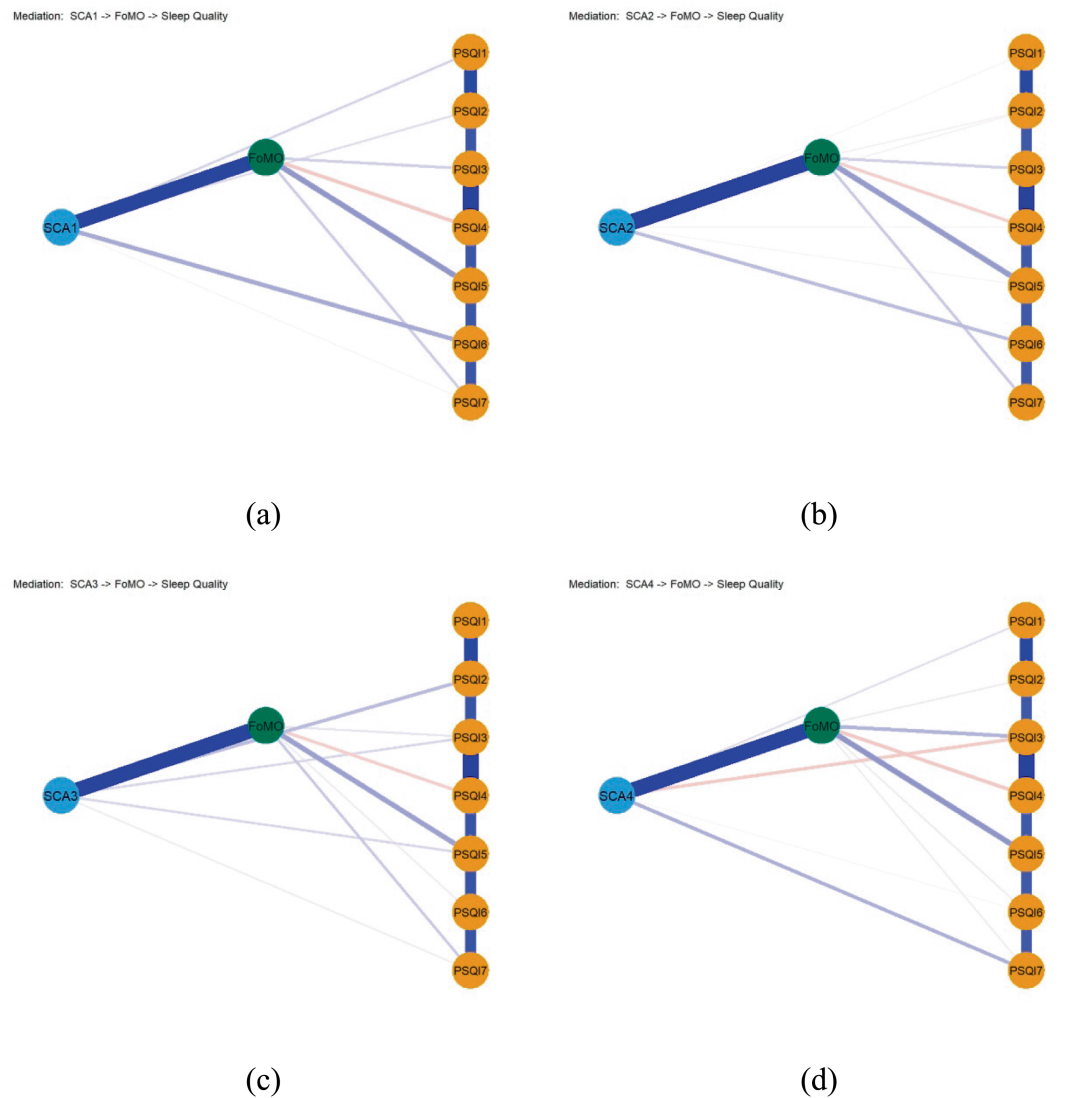
Standardized path coefficients for the mediation models illustrating the relationships between the four dimensions of short video addiction, fear of missing out, and sleep quality components. **(a)** Loss of control, **(b)** withdrawal, **(c)** escapism, and **(d)** inefficiency as independent variables.

To further examine the mechanisms underlying the relationship between short video addiction and sleep quality, four separate path models were constructed with FoMO as the mediator. The standardized results of these direct and indirect pathways are illustrated in Figure 5.

Figures 5a,b demonstrate that FoMO serves as a critical intermediary link between the loss of control and withdrawal dimensions of addiction and specific sleep outcomes. Specifically, both loss of control and withdrawal predicted heightened levels of FoMO, which subsequently associated with shorter sleep duration, improved habitual sleep efficiency, and increased daytime dysfunction. Direct pathways were also identified, where loss of control and withdrawal directly impaired subjective sleep quality, increased sleep latency, and promoted the use of sleeping medication.

As shown in Figure 5c, FoMO partially mediated the influence of escapism-oriented addiction on sleep duration, sleep disturbances, and daytime dysfunction. Escapism not only directly predicted these three sleep components but also exacerbated them through the indirect psychological bridge of FoMO, suggesting that the desire to escape reality via short videos significantly intensifies sleep interference through increased anxiety about missing rewarding experiences.

Figure 5d reveals a more complex relationship involving the inefficiency dimension. Inefficiency exerted a direct negative effect on the PSQI sleep duration score, indicating that students with higher levels of addiction-related inefficiency paradoxically tended to have longer sleep durations in the absence of other factors. However, an indirect positive effect emerged through FoMO, where inefficiency significantly increased FoMO levels, which in turn led to a substantial reduction in sleep duration. These findings suggest that while inefficiency itself might allow for longer periods of rest, the resulting fear of missing out acts as a counteracting mechanism that ultimately drives individuals to sacrifice sleep.

### 3.6 Centrality and bridge strength analysis

**Figure 6:**
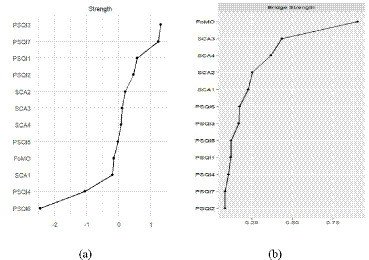
Strength centrality and bridge strength of nodes within the symptom network comprising short video addiction, fear of missing out, and sleep quality. **(a)** Strength centrality plot **(b)** Bridge strength plot.

To further quantify the structural importance of individual nodes within the psychological network, indices for strength centrality and bridge strength were computed and are visualized in Figure 6. As shown in Figure 6a, sleep duration (PSQI 3) emerged as the node with the highest strength centrality, suggesting that it represents the most influential component within the sleep quality framework. Substantial centrality values were also observed for daytime dysfunction (PSQI 7) and subjective sleep quality (PSQI 1), identifying them as prominent indicators in the network. Regarding short video addiction symptoms, withdrawal displayed the greatest strength centrality, which highlights it as a core feature of addictive tendencies among college students.

The analysis of bridge strength, presented in Figure 6b, evaluates the capacity of nodes to facilitate cross-domain connections between distinct symptom clusters. The results indicate that FoMO possessed the highest bridge strength in the entire network system. This finding confirms the critical function of FoMO as a primary psychological bridge that interlinks short video addiction dimensions with multidimensional sleep disturbances. Nodes with high bridge strength are considered essential targets for clinical intervention, as targeting FoMO may effectively deactivate the pathways through which addictive behaviors disrupt sleep hygiene.

### 3.7 Network stability and accuracy estimation

To ensure the robustness and replicability of the estimated network, the researchers performed stability and accuracy tests using the case-dropping bootstrap procedure. Given the presence of negative edges in the current topology, Expected Influence (EI) was calculated instead of traditional Strength to more accurately reflect the cumulative influence of nodes by accounting for the sign of their associations.

**Figure 7:**
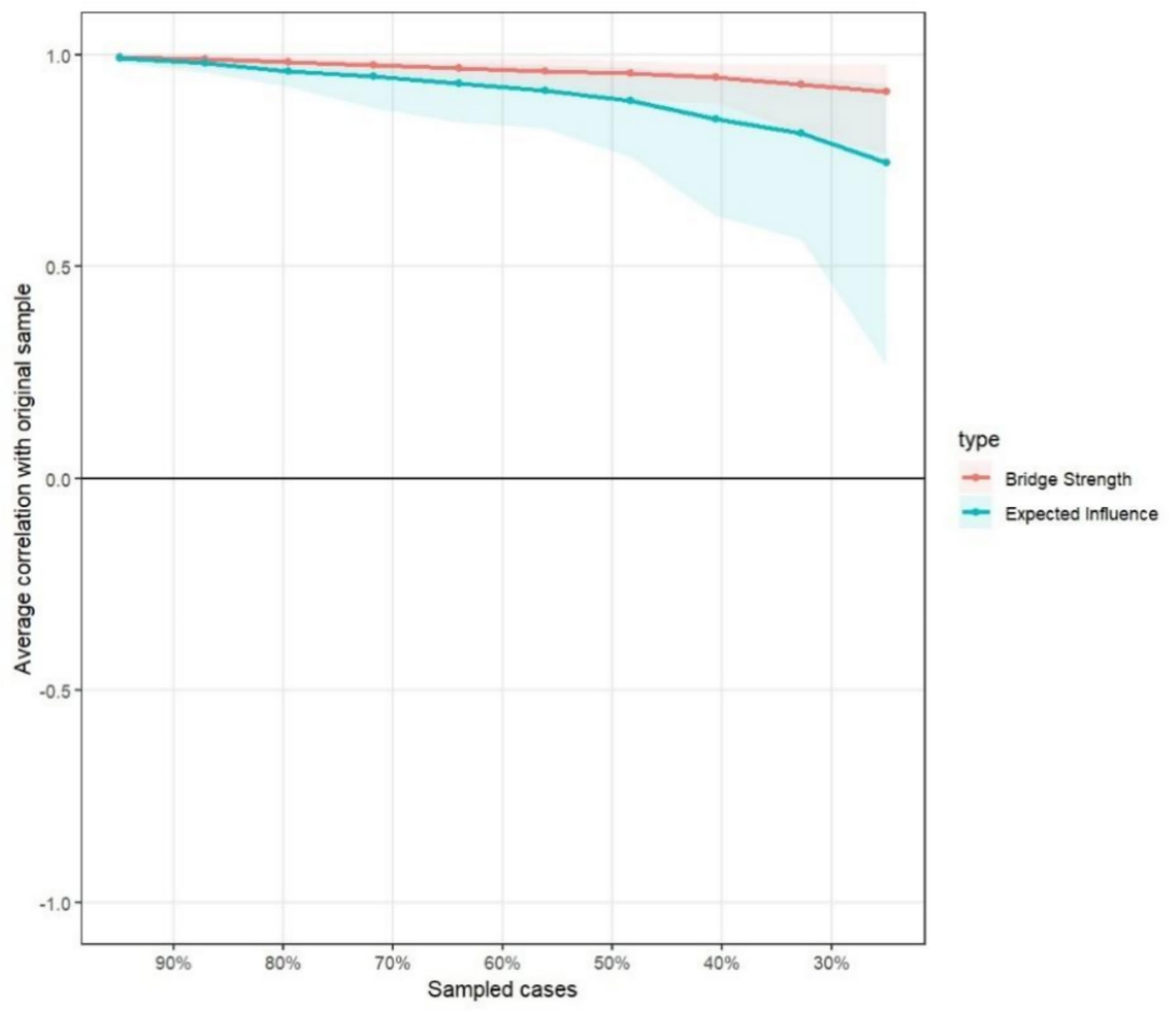
Stability of Expected Influence and bridge strength within the short video addiction, fear of missing out, and sleep quality network.

The stability results, which are presented in Figure 7, demonstrated that the Correlation Stability coefficients (CS-coefficients) were 0.594 for Expected Influence and 0.75 for Bridge Strength. According to the rigorous methodological criteria established in the literature, a CS-coefficient should ideally exceed 0.5 to ensure that the order of centrality indices remains stable even after dropping a substantial proportion of the sample. As both metrics in this study significantly surpassed this conservative threshold, the centrality and bridge strength estimations can be considered highly stable and provide a reliable basis for substantive interpretation.

## 4 Discussion

### 4.1 Demographic and behavioral correlates of sleep quality

The initial analyses of demographic differences provide an essential baseline for understanding sleep health among college students. The finding that female students reported significantly higher PSQI scores is consistent with prior research indicating greater sensitivity to digital social stressors and a higher propensity for cognitive rumination among females (29, 30). This gender disparity may be partly associated with heightened social comparison and emotional reactivity within online environments, where concerns about social evaluation are more salient. Such affective vulnerability is related to increased pre-sleep cognitive arousal and difficulties in the parasympathetic activation required for effective sleep initiation (32).

In addition, the observed peak in sleep disturbances during the junior year reflects context-specific developmental and academic pressures within the Chinese higher education system. At this stage, students typically face the combined demands of professional internships and preparation for highly competitive postgraduate entrance examinations (33, 34). This period represents a convergence of heightened performance expectations and uncertainty regarding future career trajectories, coinciding with sustained psychological stress and destabilized sleep–wake rhythms. The substantial cognitive and emotional investment required during this academic transition often occurs alongside a lack of adequate restorative sleep, marking the junior year as a critical period of vulnerability to stress-related insomnia.

Notably, patterns of digital engagement—including longer daily viewing duration and interactive behaviors such as liking and saving content—were significantly associated with poorer sleep outcomes, suggesting that active engagement may be more strongly linked to poorer sleep than passive consumption (31, 35). Unlike passive scrolling, interactive behaviors demand greater cognitive involvement and motor coordination, which are theoretically connected to intensified dopaminergic reward processing and prolonged neural activation. This sustained arousal may co-occur with sympathetic nervous system dominance beyond device use, potentially relating to delayed sleep onset and compressed total sleep duration. Collectively, the interaction between demographic susceptibility and specific digital usage patterns delineates a high-risk profile for sleep deprivation within the contemporary university population.

### 4.2 The mediating role of FoMO in digital addiction

The mediation analysis elucidates the psychological mechanisms through which short-video addiction is related to disrupted sleep architecture, identifying FoMO as a key statistical intermediary in this association (36, 37). Within the framework of self-determination theory, the compulsive monitoring of short-video updates can be conceptually linked to unmet social relatedness needs (17, 38). Although short-video platforms provide an illusion of social connection, their algorithm-driven design is associated with only partial and transient satisfaction of these needs, thereby correlating with a state of chronic psychological dissatisfaction. This unmet need state is closely associated with elevated FoMO, which operates as a form of social hypervigilance characterized by persistent concern about unobserved rewarding experiences within one’s digital network (39, 40).

Elevated FoMO is subsequently linked to heightened pre-sleep cognitive arousal and is correlated with habitual bedtime procrastination (41, 42). Pre-sleep cognitive arousal is marked by repetitive, intrusive thoughts related to social interactions and online content, which are associated with difficulties in achieving the neural deactivation necessary for sleep initiation. This process is frequently accompanied by impaired self-regulatory capacity, often corresponding to individuals intentionally delaying sleep despite awareness of its adverse consequences. In this context, digital devices function as short-term anxiolytic tools that temporarily relieve social unease while paradoxically co-occurring with sustained elevated levels of mental alertness.

In addition, the observed direct pathways linking loss of control and withdrawal symptoms to prolonged sleep latency and increased use of sleep medication underscore the profound association between behavioral addiction and the disruption of biological sleep imperatives. These addiction dimensions reflect severe dysregulation of sleep–wake homeostasis, whereby the immediate reinforcement of digital consumption appears to take precedence over the physiological drive for rest (43, 44). When withdrawal symptoms or complete loss of behavioral control emerge, individuals may increasingly rely on pharmacological aids to manage insomnia, signaling substantial disruption of circadian regulation. Collectively, the interaction between psychological anxiety and behavioral dysregulation is indicative of a chronic sleep debt that is linked to significant risks to the long-term health of university students.

### 4.3 Topological importance of sleep duration and FoMO

The application of network analysis provides a fine-grained representation of symptom architecture by conceptualizing addictive behaviors and sleep outcomes as an interrelated system rather than independent variables. The high strength centrality of sleep duration (PSQI-3) identifies it as a core hub within the sleep disturbance network, indicating that variations in this node are particularly likely to co-occur with widespread structural changes across the broader sleep architecture (45, 46). As a fundamental marker of physiological recovery, sleep duration functions as a critical bottleneck for overall sleep health, as its reduction often constitutes the most immediate correlate of time displacement associated with excessive digital engagement. Deficits in this central node frequently coincide with broader effects, including daytime dysfunction and reduced subjective sleep satisfaction, thereby correlating with chronic sleep debt.

Bridge strength analysis further demonstrates that FoMO occupies a strategic topological position as the principal conduit linking the affective dimensions of addiction to physiological sleep processes (47, 48). While short-video addiction reflects a behavioral pattern, FoMO supplies the emotional urgency that connects psychological craving with nocturnal sleep disruption. In this sense, FoMO operates as a gateway symptom that maintains structural connectivity between the addiction symptom cluster and the sleep disturbance cluster. Absent this bridge, excessive short-video use may remain a habitual behavior without necessarily being accompanied by clinically significant sleep impairment.

These structural insights carry important clinical implications. Interventions that specifically target the deactivation of this bridge symptom may yield greater efficacy than traditional approaches that focus solely on reducing overall screen time or addressing addiction symptoms in isolation (25, 49). By attenuating anxiety related to perceived social disconnection, clinicians may effectively address the association between adaptive digital behaviors and their subsequent sleep-related consequences, offering a more precise and mechanism-oriented pathway for intervention.

### 4.4 The paradox of inefficiency and suppression effects

The present findings reveal a particularly nuanced role of the inefficiency dimension of short-video addiction, manifested as a statistically significant suppression effect (50). Although inefficiency was directly associated with longer sleep duration, its indirect statistical path involving fear of missing out (FoMO) was strongly positive, ultimately correlating with poorer overall sleep quality (51). This paradoxical pattern suggests that behavioral sluggishness or reduced daytime productivity may initially coincide with greater opportunities for physical rest. However, this apparent benefit is frequently accompanied by heightened psychological distress, including feelings of guilt and perceived social underachievement (52, 53).

When individuals perceive themselves as unproductive during the day, they may experience an increased compulsion to monitor social dynamics during nighttime hours as a means of alleviating anxiety related to falling behind peers. Elevated FoMO thus acts as a countervailing force, closely linked to compensatory nocturnal digital engagement that corresponds to a reduction in the sleep opportunity theoretically associated with daytime inactivity. This pattern reflects a form of nocturnal social compensation, in which the pursuit of belonging and agency appears to take precedence over homeostatic sleep drive.

Collectively, these results underscore a dynamic tension between behavioral passivity and cognitively driven anxiety associated with excessive digital consumption (54, 55). The psychological burden of perceived daytime inefficiency is related to a heightened need for late-night social validation, indicating that the negative associations between short-video addiction and sleep extend beyond simple time displacement. Rather, sleep disruption is embedded within a complex associative network in which daytime behavioral deficits correspond with elevated emotional anxieties that are in turn linked to poorer nocturnal recovery. This suppression mechanism highlights the importance of interventions that address not only maladaptive usage patterns but also the underlying feelings of inadequacy and the compensatory motivations that are associated with nocturnal digital engagement.

### 4.5 Practical implications and limitations

From a practical standpoint, the present findings suggest that institutional mental health interventions should move beyond simplistic screen-time restrictions, which often fail to address the compulsive and psychologically reinforced nature of digital engagement. Instead, prevention and intervention strategies should target the latent psychological mechanisms that are linked to the maintenance of addictive behaviors, with particular emphasis on reducing FoMO through structured cognitive-behavioral approaches. Universities may consider integrating digital literacy programs that enhance students’ awareness of algorithm-driven persuasive design while encouraging adaptive disengagement strategies, such as cultivating the “joy of missing out,” which emphasizes intentional detachment from digitally mediated social pressures.

In addition, mindfulness-based stress reduction interventions could be adapted to specifically address pre-sleep cognitive arousal and autonomic hyperactivation that may be linked to excessive short-video use. By promoting attentional regulation and emotional decoupling from online social comparison, such interventions may help reduce compulsive digital consumption patterns and may contribute to better sleep hygiene among university students. However, these practical implications should be interpreted cautiously. Although symptoms with higher centrality or bridge strength may indicate potentially important targets for future intervention research, network centrality does not by itself demonstrate that modifying these symptoms will necessarily lead to superior intervention outcomes.

Several methodological limitations warrant cautious interpretation of the findings. First, the cross-sectional design precludes definitive causal inference; therefore, the observed relationships must be interpreted strictly as statistical associations rather than causal pathways, although the proposed models are theoretically grounded and empirically supported. Second, the reliance on self-report measures may introduce social desirability bias and retrospective recall errors related to sleep quality and digital media use. Although the Pittsburgh Sleep Quality Index is a well-validated instrument, it may not fully capture objective sleep parameters as assessed by actigraphy or polysomnography. Moreover, because all variables were collected using self-report questionnaires at a single time point, the findings may also be affected by common-method bias. Third, the sample was drawn exclusively from universities in southern China, which may limit the generalizability of the findings to populations with differing cultural norms, academic demands, or digital media environments. Fourth, although several demographic variables were statistically controlled in the mediation analyses, some potentially important confounding variables, such as baseline anxiety, chronotype, and academic stress, were not directly measured in the present study and may have influenced the observed associations. Finally, because sleep quality was assessed exclusively through self-report, the present findings should be interpreted as reflecting perceived sleep problems rather than objectively verified sleep parameters.

Future research should employ longitudinal designs to examine the temporal stability and causal dynamics of symptom networks linking digital addiction, FoMO, and sleep disturbances. The application of ecological momentary assessment methods would enable more fine-grained analysis of real-time interactions between short-video engagement and sleep-related processes in naturalistic settings. Moreover, integrating objective sleep-tracking technologies with psychological assessments would provide greater insight into the physiological mechanisms through which FoMO influences sleep architecture. Finally, several methodological limitations warrant cautious interpretation of our findings. Given the cross-sectional design of the current study, the observed relationships represent robust statistical associations rather than definitive causal pathways. While our theoretical framework is empirically supported, future longitudinal or experimental designs are essential to elucidate the directional causality and temporal stability of these mechanisms.

## 5 Conclusion

The present study offers an empirically grounded framework for elucidating the complex interplay among digital addiction, psychological anxiety, and sleep architecture. By integrating traditional mediation analysis with network-based topology, the findings identify fear of missing out (FoMO) as a critical psychological conduit through which maladaptive digital behaviors translate into physiological sleep impairment. Specifically, sleep duration emerged as a central hub within the sleep disturbance network, while FoMO functioned as the primary bridge node linking short-video addiction symptom clusters to multidimensional sleep disturbances.

Importantly, the observed suppression effect associated with the inefficiency dimension highlights a paradoxical dynamic between behavioral passivity and compensatory nocturnal anxiety induced by excessive digital engagement. Although behavioral inefficiency was directly associated with longer sleep duration, its indirect effect via FoMO ultimately contributed to sleep curtailment. This pattern suggests that the detrimental impact of short-video addiction on sleep quality is driven less by mere screen exposure and more by an emotionally driven need for social validation that sustains pre-sleep cognitive arousal and counteracts biological sleep pressure.

Collectively, these findings indicate that sleep health in the digital era is shaped not only by the quantity of media use but also by the psychological processes that maintain heightened arousal during the pre-sleep period. From an applied perspective, the centrality of bridge symptoms underscores the limitations of time-restriction–based interventions. Mental health strategies within university settings should instead prioritize the identification and deactivation of key psychological bridges, particularly FoMO, through targeted cognitive-behavioral interventions and digital literacy programs. Addressing the cognitive–emotional mechanisms underlying digital dependency may therefore represent a more effective approach to mitigating the systemic impact of short-video addiction on student sleep health and overall well-being.

## Data Availability

The original contributions presented in the study are included in the article/supplementary material, further inquiries can be directed to the corresponding author.
